# Middlesex Hospital

**Published:** 1832-06

**Authors:** 


### HOSPITAL REPORTS.

### MIDDLESEX HOSPITAL.

*Efficacy of Iodine in some Affections of the Skin of Syphilitic Origin.* By Herbert Mayo, F.R.S.

During the last half year, Mr. Mayo has employed iodine in a variety of chronic diseases. The formula in which he has prescribed the remedy is the following: ℞. Tr. Iodine, ʒi.; Hydriod. Potassæ, ʒss.; Syrupi Papaveris, ℥ss.; Aquæ Menthæ Viridis, ℥viij. ad ℥x. This quantity to be taken by adults during the twenty-four hours; a less quantity by children.

Mr. Mayo states, that he has not found any benefit arise from the use of this medicine in scirrhus, in strumous glandular enlargements, or in strumous affections of the joints; but that it has proved strikingly useful in cases of bronchocele, (conjoined with its local applications,) and in some affections of the skin of syphilitic origin. The two cases which follow are remarkable instances of its efficacy in the latter class of complaints.

Case I. John O'Shaugnessy, ætat. thirty-two, was admitted into the hospital in January 1831. The preceding August he had contracted a chancre with bubo, for which mercury had been administered, so as to produce ptyalism: the ulcer and bubo healed under this treatment. But, according to his very imperfect recollection of the progress of his disorder, about five weeks after he commenced the use of mercury, and while he was still taking it, his throat became ulcerated; he lost on one occasion, by a sudden hemorrhage from the throat, nearly a quart of blood. Afterwards, copper-coloured spots, followed by ulcers, appeared upon his body. He believes that the mercury was then discontinued, and that he took sarsaparilla, and that he mended upon this change.

At the period of his admission, there was a large superficial ulcer upon the instep, a great part covered with healthy granulations, but having a yellow phagedenic edge, with a narrow bright red line upon the skin around it; he had another ulcer of a similar description on one shoulder, and several large leprous spots on the limbs and body. Sarsaparilla with Liquor Potassæ was now given to him; and, while taking this medicine, various local applications being employed at the same time, the ulcer upon the instep healed: but the malady nevertheless gained ground; he gradually lost strength, and his skin became covered with blotches and ulcers. The ordinary character of the affection at its commencement was that of Lepra Alphoïdes; each spot, however, soon became tubercular, and, in place of the thickened white patch of cuticle, a crust formed, below which was an ulcer. There are many of these ulcers on the limbs and body, and the forehead and face were covered with them; the lips, the alæ of the nose, the eyebrows, were equally involved in ulcerated blotches: he suffered from burning heat of the body and face; and the ulceration of the lips produced profuse ptyalism. The bones were not affected; but there was pain on moving the joints of the legs, and a small depôt of serous fluid formed in the calf of the right leg.

While this patient's sufferings became thus aggravated, a variety of remedies were tried in succession. Sarsaparilla with liquor arsenicalis, and with the oxymuriate of mercury; the decoction of the Smilax aspera; the bluepill, so as slightly to touch the gums; bark, with the nitric and muriatic acids; the strong nitric acid, in doses of a drachm a day: all of these remedies, in their turn, were observed to be of a slight but temporary benefit, and to produce for a fortnight after the first trial a perceptible improvement, after which the patient fell back again; with the exception of the bluepill, and the strong nitric acid: both of these were discontinued in ten days after commencing their use, no amendment, but rather an aggravation of the symptoms, having ensued.

And thus the patient went on from January 1831, to the beginning of December, the disease steadily gaining ground, when at M. Magendie's suggestion, Mr. Mayo prescribed iodine.

The effect of this remedy, after a very few day's use, was very striking; the skin became less red and heated, several of the crusts separated, and the ulcers put on a healthier appearance. The remedy has been persevered in, with some interruptions, up to the present time, and the patient is now all but entirely recovered.

The use of this medicine has, however, been interrupted on four different occasions for a fortnight; the march of amendment having been the following. For three to four weeks consecutively there has been progressive improvement; then the patient has fallen back a little, or become stationary. The medicine has then been discontinued; the disease has then uniformly become aggravated; upon which, at the expiration of about a fortnight, the medicine has been resumed; and on each occasion has been signally and immediately beneficial. At each relapse, the patient has fallen back to a less degree than at the preceding.

Case II. John Saxton, ætat. thirty-one, came under Mr. Mayo's care, after having suffered a great length of time with ulcerated throat and face, and pains and tenderness of the head; a train of symptoms which began in a venereal sore and a course of mercury.

It is now two years since the scalp upon the forehead became at two points puffy and tender, but not discoloured; it soon became red, and broke; and then, for many months, his face was disfigured in the following manner: two or three ulcers formed on the forehead, eyebrows, and bridge of the nose, round which the skin was red and thickened; and these spread after healing where they began, while they extended over a fresh surface. With this patient, while in this state, every remedy enumerated as having failed in the preceding case was tried, and each change was temporarily useful. The Decoctum Smilacis Asperæ, at one time, and a mild mercurial course at another, produced more decided benefit, however, than the other remedies which were used. This patient began the use of iodine at the same time with O'Shaugnessy. He has entirely recovered; the ulcers having gradually healed, and the adjacent skin having lost in a great degree the redness and tumefaction.

